# The Medical Inspection of Schools

**Published:** 1903-02-14

**Authors:** 


### The Medical Inspection of Schools.

When those who are responsible for the education of the young, and especially those to whom is entrusted the conduct of that "elementary" education which is now compulsory upon all, once become thoroughly awake to the serious nature of the charge which they have undertaken, we may expect the medical problems involved to receive much more attention than has so far been accorded to them. The duties of the public in regard to children compulsorily removed from the care of their parents extend far beyond the provision of educational facilities. So far as school hours are concerned the public authority stands *in loco parentis*, and under such circumstances it is obvious that questions of health become matters of first importance.

At the present moment a certain degree of awakening of the public conscience in regard to this matter is taking place; here and there school authorities are appointing medical officers who, in addition to giving general advice on health questions, are supposed to undertake some sort of medical inspection of the schools if not of the scholars, and the very fact that such a beginning is being made renders it desirable before new ways get fixed and crystallized and new vested interests become interwoven in the system, to urge that the whole matter of the medical inspection of schools and scholars touches so intimately upon matters of public health, that instead of the school managers engaging their own inspectors and instead of these inspectors having regard merely to what is good and advisable from an educational point of view, the medical inspectors of schools ought to be appointed by the sanitary authorities, and ought in their inspections to have regard to what is best for the good of the community. The time is peculiarly appropriate for the discussion of this matter, since under the new Education Act a definite link is provided between the sanitary and educational departments of the local authorities of the country.

In comparison with the very feeble efforts made to discover the beginnings of illness in schools even by the most progressive school managers in this country let us consider what is regarded in New York or Boston as an essential step for the prevention, or at least for the diminution of the dangers inseparable from school life. In New York City 250 medical inspectors are daily engaged in the work. This medical inspection is done under the Sanitary Bureau of the Department of Health, and is made to apply not only to the public schools but also to parochial and other schools managed by various bodies and societies. In Boston there are 50 inspectors who are authorised agents of the Board of Health. In addition to visiting the public schools daily to examine all sick pupils and to advise the teachers in regard to them, they also decide such questions as may arise as to the propriety of excluding from the school other children living in the same building, and visit every reported case of diphtheria, membranous croup, and scarlet fever to ascertain the effectiveness of the isolation.

The system appears to be much the same wherever it is put in practice. The inspection is made in the early school hours, generally between 9 and 10 A.M., the teachers referring to the examiner all such pupils as present any symptoms of illness. Tne examiner occupies a separate room in which he investigates the cases one by one, makes his diagnosis, and decides what steps are to be taken. In all cases of infectious disease not only is the child at once sent home but immediate notification of the case is given to the sanitary office. Moreover, in these infectious cases the physician examines all the members of the same class or those who have been sitting near the affected pupil, taking cultures from the throat if necessary, so as to pick out any who may have become infected at the earliest possible moment.

That the medical inspection of schools so carried out is likely to prove of the greatest possible service to the community we can have no doubt, while it seems equally clear that it is only just to those children who are compelled by law to expose themselves to the risks of school life, that everything that is possible should be done to reduce those risks to the smallest possible dimensions. The point however on which we would just now more particularly insist is that all this medical inspection is a matter of public health rather than of education, and that it should be definitely handed over to the public health authorities.

